# Numerical Investigation of the Seismic Performance of Steel Frames with Energy-Dissipating Composite Walls

**DOI:** 10.3390/ma15030828

**Published:** 2022-01-21

**Authors:** Ding Wei, Jia Suizi

**Affiliations:** School of Engineering and Technology, China University of Geosciences, No. 29, Xueyuan Road, Haidian District, Beijing 100083, China; 3002210001@email.cugb.edu.cn

**Keywords:** steel frame structure, replaceable energy dissipation structure, seismic performance, numerical simulation, rural house

## Abstract

To improve the seismic performance of steel frame buildings in rural areas, an energy-dissipating composite wall (EDCW) assembled from concrete-filled steel tubular columns and concrete sheet walls was designed. Cyclic loading tests were simulated using the finite element method (FEM) to analyse the seismic performance of the EDCW. The reliability of numerical modelling and analysis was verified by comparing the hysteretic curves obtained by the finite element model with those obtained by previous experiments. The EDCW was designed for installation in a two-storey steel frame, and the FEM was used to determine the seismic performance of the steel frame, including the deformation and failure characteristics, hysteresis curves, and skeleton curves. The numerical simulation results showed that the EDCW dissipated most of the seismic energy and thus substantially improved the seismic performance of the frame. The seismic performances of 16 frames were compared to investigate the effects of the span ratio of the steel frame to the EDCW, the installation location of the EDCW, and the stiffness of the steel frame on the seismic performance of the frame.

## 1. Introduction

Earthquakes are common disasters that damage houses. In rural areas of China, brick wood structures and brick concrete structures are the most common forms of dwelling structures. Due to their poor seismic performance, many rural houses are damaged or destroyed in earthquake-stricken areas, causing enormous economic losses [1,2]. Steel frame structures [3,4,5,6] have the advantages of a large building space, flexible layout, excellent seismic performance, and ease of construction and thus have been widely used in the reconstruction of rural houses after earthquakes.

To further improve the seismic performance and reduce the cost of post-disaster reconstruction, components with a high energy dissipation capacity and that can be easily replaced when damaged have begun to be installed in steel frame structures [7,8,9,10,11,12,13,14,15,16,17].

Replaceable steel coupling beams are a common type of replaceable energy dissipation component. Through a series of experimental comparisons, Lu et al. found that shear walls with replaceable coupling beams had a high energy dissipation capacity and low strength degradation, and the damage locations of the coupling beams were concentrated in the energy-dissipating sections of the bolted connections, facilitating replacement after an earthquake [18,19,20,21]. Ji et al. proposed a similar replaceable steel coupling beam and applied it to coupled shear wall structures [22,23,24]. Shen et al. developed a type of plastic replaceable link and connected these links to the ends of beams close to the beam-to-column joints [23]. Mansour et al. designed two forms of replaceable coupling beam sections for eccentrically braced frame structures [24]. Fortney et al. installed a shear steel fuse in the middle portion of a steel coupling beam [25]. Lyons et al. proposed a device that formed a ductile fuse through a connection in a series with a viscoelastic damper in the coupling beam of the reinforced concrete shear wall [26]. Christopoulos et al. proposed the use of a viscoelastic coupling damper [27]. Wang proposed the use of a metallic damper for coupling beams [28,29]. The results from the above studies all showed that the dampers for coupling beams had an excellent energy dissipation capacity. In addition, Volynkin et al. found that the replaceable coupling beam welded only at the flange had a higher rotational capacity than that welded at the web and flange, but this form of construction did not change the ultimate failure mode of the replaceable coupling beam [30].

Replaceable shear wall corner components are another type of replaceable energy dissipation device. Jiang et al. proposed a replaceable wall corner component with a mild steel yielding damper, and they showed that shear walls installed with these components could direct the damage to replaceable components, thereby protecting the nonreplaceable areas from damage [31]. Considering the susceptibility of shear wall corners to damage, Lu proposed a new type of shear wall with replaceable corner components by installing replaceable tension-compression bearings at the corners of the shear wall, and these bearings dissipated energy during earthquakes and could be replaced after earthquakes [32].

The third type of a replaceable energy dissipation device is the steel plate wall. Cortés and Liu found that a vertically slitted steel plate wall-frame structure exhibited better energy dissipation characteristics than ordinary shear walls, and the damage always occurred as plastic damage to the connection area between the vertical slits of the steel plate wall. Additionally, bolt-connected steel plate walls can be prefabricated, field assembled, and replaced over time [33]. A shear wall structure with replaceable steel plate walls was proposed at the U.S. Multidisciplinary Research Centre for Earthquake Engineering, and a full-scale model test of a two-story steel plate shear wall was conducted [31]. The results showed that the replaceable steel plate walls exhibited good buckling and energy dissipation characteristics, and the hysteretic performance of the structure after steel plate replacement was essentially the same as that before replacement [34].

To improve the seismic performance of rural houses and reduce the costs of post-disaster reconstruction, an energy-dissipating composite wall (EDCW) assembled from concrete-filled steel tubular columns (CFSTCs) and concrete sheet walls. First, cyclic loading tests on the EDCW were reproduced using numerical simulations to analyse the seismic performance of the EDCW. Then, the EDCW was installed in a two-storey steel frame, and the seismic performance of the steel frame was analysed using numerical simulations. Finally, the seismic performances of 16 frames were compared to investigate the effects of the span ratio of the steel frame to the EDCW, the installation location of the EDCW, and the stiffness of the steel frame on the seismic performance of the frame.

## 2. Steel Frame with EDCWs

### 2.1. Structural Composition

**Figure 1:**
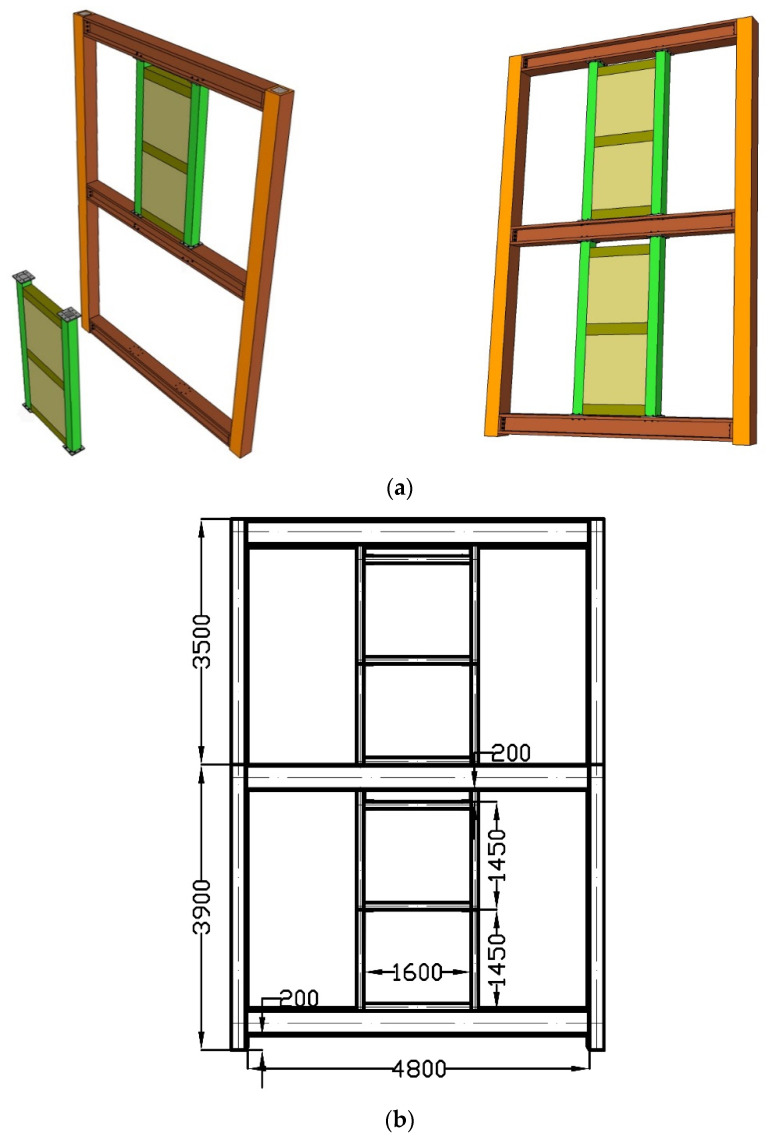
Steel frame structure with EDCWs. (**a**) Assembly; (**b**) structure diagram (Unit: mm).

To improve the seismic performance of steel frame structures, the present study proposes a new steel frame structure with replaceable EDCWs for rural residential buildings. This type of steel frame structure applies to rural building structures with three stories or fewer, a storey height of no more than 4 m, and a total height not exceeding 10 m. The steel frame composite structure (Figure 1a) is composed of an external steel frame structure and EDCWs. The EDCWs consist of a CFSTC frame and recycled concrete sheet wall. The geometries of the steel frame structure are shown in Figure 1b. The EDCWs mainly resist the horizontal seismic load, and they are installed between the upper and lower frame beams, close to frame columns or door and window openings. The boundary components of the EDCWs are connected by high-strength bolts with the upper and lower I-beams of the steel frame of the rural house.

### 2.2. Composition of the EDCW

**Figure 2:**
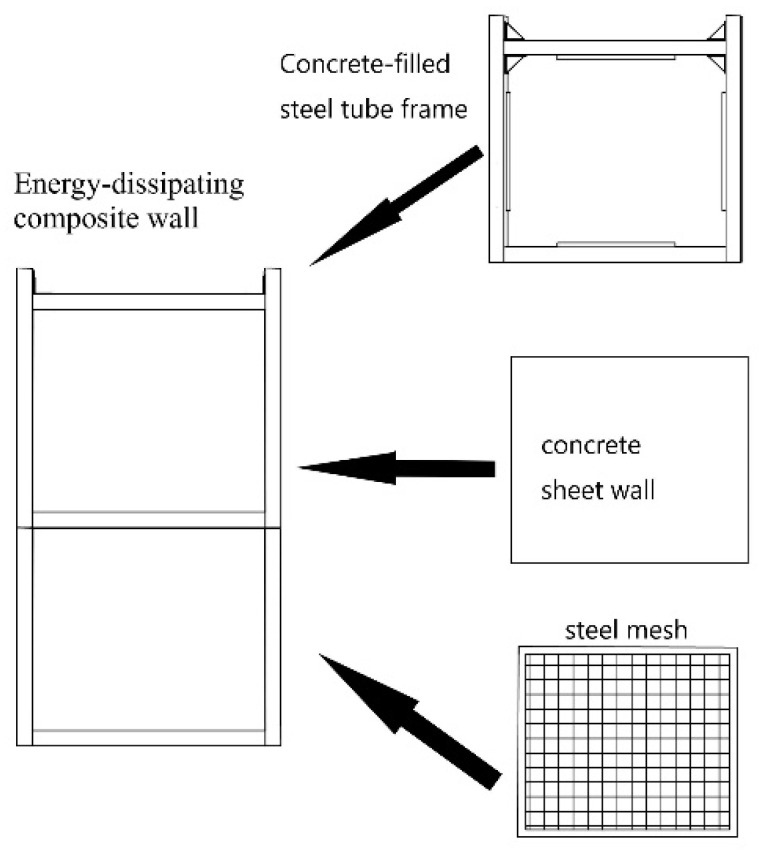
Composition of the EDCW.

The replaceable EDCW is composed of CFSTCs and assembled concrete sheet walls with steel mesh (Figure 2) fitted into the steel frame (Figure 1). The boundary components of the EDCW are made of CFSTCs and beams connected through reinforced joints. The dimensions of the components are shown in Figure 1b, and the material parameters are listed in Table 1. The concrete wall panel is made of concrete cast with 4-mm-thick, 40-mm-wide frame steel plate strips and a bidirectional distribution of 5-mm-diameter rebars. The concrete wall has a thickness of 60 mm, and its material parameters are provided in Table 2.

### 2.3. Structural Composition of the Steel Frame

The steel frame consists of CFSTCs and I-beams welded together and is the main structure that carries the vertical load of the house. Table 3 lists the cross-sectional dimensions and material mechanical parameters of the CFSTCs and I-beams. The mechanical properties of the recycled concrete inside the steel pipes are shown in Table 2.

## 3. Numerical Tests

Large full-scale cyclic loading tests on structures are expensive and difficult to implement. In contrast, numerical simulations have the advantages of low cost, high efficiency, strong adaptability, and repeatability [18]. Therefore, the present study used the finite element software Abaqus to simulate cyclic loading tests on EDCWs and steel frame structures with EDCWs to analyse their seismic performances.

### 3.1. Calculation Models

**Figure 3:**
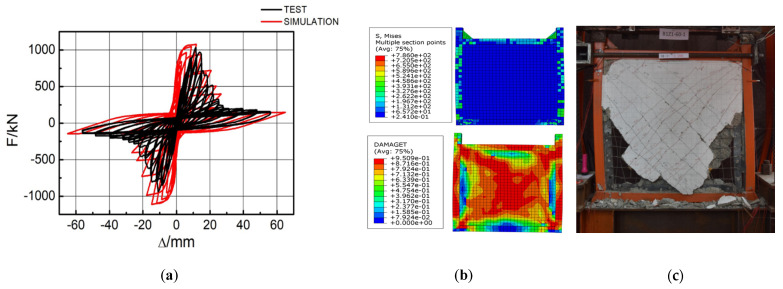
Comparison of simulation and literature 35. (**a**) Comparison between experimental curves and simulated curves. (**b**) Stress contour plots; (**c**) experimental damage.

The research group previously carried out a preliminary low-cyclic load test on EDCWs [35]. The simulated hysteretic curve was compared with the experimental hysteretic curve, and the simulated peak load value was 4% greater than the experimental value. The variation trend of the two hysteretic curves was consistent, showing a butterfly shape (Figure 3a). Two seismic defence lines were apparent, dissipating energy in stages.

It can be seen from Figure 3b that the center stress of the wall panel of EDCW is larger along the diagonal direction. Consistent with the phenomenon in the test, visible vertical cracks appeared at the connection between the wall panel and steel tube column. With continuous loading, vertical cracks expand and extend upward, forming dense fine cracks; diagonal cracks occur in the wallboard along the main diagonal direction. It is roughly consistent with the experimental phenomenon. After the concrete wall panel is out of use, the outer frame is obviously bent and deformed at the column foot. Comparing EDCW simulation results with the previous test results enabled the use of Abaqus finite element software for seismic component modelling analysis in this paper.

The Abaqus modelling tool (Dassault Systems Simulia Corp, Providence, RI, USA) [36] was used to construct the computational model of the EDCW and steel frame with the EDCWs (Figure 3). The square steel tubes of the boundary beams and columns, the concrete inside the steel tubes, and the concrete wall panels of the EDCW were modelled using three-dimensional (3D) linear integral elements; the rebars inside the wall panels were modelled using 3D two-node truss elements; and the connecting steel plates and steel plate strips inside the wall panels were modelled using four-node general-purpose shell elements.

**Figure 4:**
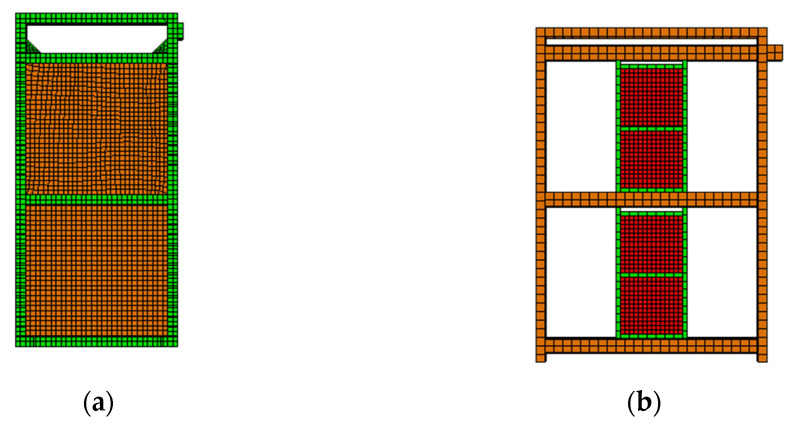
Computation models. (**a**) Computation model of the EDCW; (**b**) computation model of the steel frame.

To evenly distribute the vertical load to the CFSTCs on both sides, a loading beam was added above the boundary beam at the top of the EDCW, and a loading head was mounted at the top right of the EDCW to avoid stress concentration of the horizontal load at the loading position (Figure 4a). The boundary steel columns and I-beams were modelled using 3D linear integral elements, and the EDCW was connected directly with the I-beams using “Tie”.

To evenly distribute the vertical loads to the boundary steel tubes on both sides, a loading beam was added at the top of the steel frame, and a loading head was mounted at the top right of the steel frame (Figure 4b).

### 3.2. Loading Method

(1) Loading method for the EDCW

**Figure 5:**
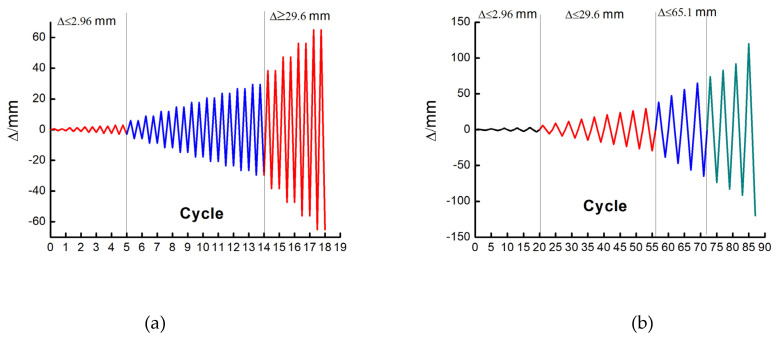
Loading protocols. (**a**) Loading protocol for the EDCW; (**b**) loading protocol for the steel frames.

A vertical load of 600 kN was applied, and a horizontal cyclic load was applied laterally to the top of the boundary column of the EDCW. Displacement control was used for the horizontal loading with the following loading protocol (Figure 5a): The increment of the drift ratio was set to 1/2500 until the drift ratio reached 1/500; then, it was set to 1/500 until the drift ratio reached 1/50; subsequently, it was set to 3/500. Two cycles occurred at each level of loading. The test ended when the specimen obviously failed, the loading could not be continued, or the horizontal load dropped below 85% of the peak load.

(2) Loading method for the frame structure

A vertical load of 800 kN was applied, and a horizontal cyclic load was applied laterally to the top of the boundary column of the frame. Displacement control was used for horizontal loading with the following loading protocol (Figure 5b): The increment of the drift ratio was set to 1/3500 until the drift ratio reached 1/2000; then, the increment of the drift ratio increased to 1/500 until the drift ratio reached 1/200; after the drift ratio exceeded 1/30, the increment of the drift ratio further increased to 1/250. One cycle occurred at each level of loading. The test ended when the specimen obviously failed, the loading could not be continued, or the horizontal load dropped below 85% of the peak load.

### 3.3. Yield Model

(1) Plastic yield model of concrete

The concrete plastic yield model was used to reflect the plastic deformation and failure of concrete materials. The concrete plastic yield model was a continuum damage model, which adopted isotropic damage parameters as its internal variables and used the behaviour under tensile and compressive stresses to describe the inelastic deformation and failure of materials [36]. The damage indices *D_W_* and *D_F_* were used to determine the tensile and compressive failures of concrete, respectively, and they were calculated using Equations (1) and (2), respectively.

(1) $$D_{w}=1-\frac{\sigma_{t}}{E_{0}\left(\varepsilon_{t}-\varepsilon_{t}^{pl}\right)}$$

(2) $$D_{F}=1-\frac{\sigma_{c}}{E_{0}\left(\varepsilon_{c}-\varepsilon_{c}^{pl}\right)}$$

where *σ_t_* is the tensile stress, $\varepsilon_{t}$ is the tensile strain, $\varepsilon_{t}^{pl}$ is the plastic tensile strain, *E_0_* is the initial elastic modulus, *σ_c_* is the compressive stress, $\varepsilon_{c}$ is the compressive strain, and $\varepsilon_{c}^{pl}$ is the plastic compressive strain.

(2) Yield model of the steel structure

The von Mises criterion [36] was used to determine the plastic deformation and failure of the steel structure. This criterion meant that the material yielded when the stress–strain state at a certain point reached a constant value or that the equivalent stress was always a constant value when the material was in the plastic state.

## 4. Results and Discussion

### 4.1. Seismic Performance of the EDCW

(1) Process and characteristics of deformation and failure

**Figure 6:**
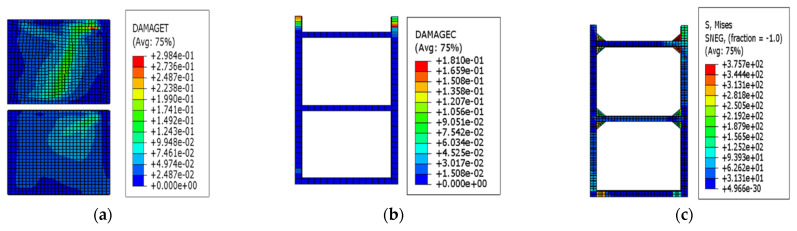
Stress contour plots at a drift ratio of 1/500. (**a**) Stress contour plot of the wall concrete; (**b**) stress contour plot of the concrete in the boundary steel tube; and (**c**) von Mises stress contour plot of the boundary steel tube.

The loading was carried out according to the loading protocol shown in Figure 5 to obtain the deformation and failure characteristics of the EDCW at different loading stages. When the drift ratio of the EDCW reached 1/500, local tensile failure occurred in the upper right part of the concrete wall, with the failure area accounting for approximately 0.53% of the whole wall area (Figure 6a). At this moment, the steel tubes and concrete inside them were in the elastic deformation stage.

“DAMAGET” and “DAMAGEC” in Figure 6 refer to the tensile damage factor and the compressive damage factor, respectively, which represent the crack propagation trend when the component is damaged in tension/compression. “Avg75%” is the default averaging threshold (default averaging threshold) amount used to average variables (usually stress), of which 75% can be modified, generally set to 75%, indicating that when the relative node variable is less than this value, the node results are averaged, where the relative node variable = (the maximum value of the node variable − the minimum value of the node variable)/(the maximum variable value of all nodes in the area − the minimum variable value of all nodes in the area). “SNEG” is defined from the element distance from the midplane to the reference plane.

**Figure 7:**
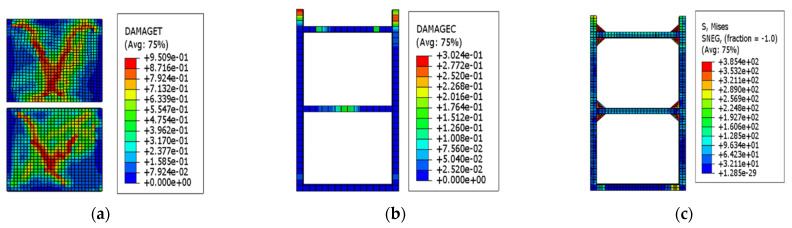
Stress contour plots at a drift ratio of 1/200. (**a**) Stress contour plot of the wall concrete; (**b**) stress contour plot of the concrete in the boundary steel tube; and (**c**) von Mises stress contour plot of the boundary steel tube.

As the drift ratio further increased, the region of the wall where concrete yielded expanded continuously. When the drift ratio of the EDCW was 1/200, the concrete failure expanded from the centre of the concrete wall to its four corners, resulting in an X-shaped failure region, with the tensile failure area accounting for approximately 43.21% of the total wall area (Figure 7a). The concrete cast in the boundary steel tube did not exhibit substantial damage (Figure 7b). The maximum von Mises equivalent stress of the boundary steel tube was 321.1 MPa (Figure 7c), which was less than the yield strength of steel.

**Figure 8:**
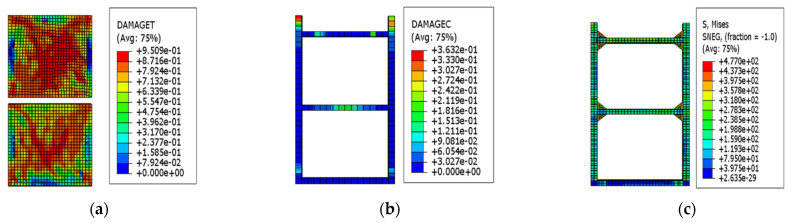
Stress contour plots at a drift ratio of 1/130. (**a**) Stress contour plot of the wall concrete; (**b**) stress contour plot of the concrete cast in the steel tubular columns; and (**c**) von Mises stress contour plot of the steel tubular columns.

When the drift ratio of the EDCW reached 1/130, most regions of the concrete wall failed completely, with the failure area accounting for approximately 73.18% of the total wall area (Figure 8a). Most regions of the concrete wall lost their bearing capacity and became ineffective. At this point, local failure occurred in the upper part of the boundary column, with the failure area accounting for approximately 0.62% of the total area (Figure 8b). The maximum von Mises equivalent stress of the boundary steel tube was 397.5 MPa (Figure 8c), which was greater than the yield strength of steel, indicating that the boundary components started to yield locally.

**Figure 9:**
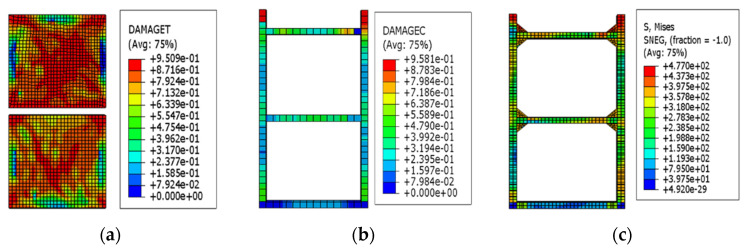
Stress contour plots at a drift ratio of 1/27. (**a**) Stress contour plot of the wall concrete; (**b**) stress contour plot of the concrete cast in the steel tubular columns; and (**c**) von Mises stress contour plot of the boundary steel tubular columns.

After the drift ratio exceeded 1/130, the load was taken entirely by the CFSTCs of the EDCW, and the damaged region of the boundary components gradually expanded. The loading was stopped at a drift ratio of 1/27, when the failure area of the concrete cast in the steel tubes accounted for approximately 46.13% of the whole area (Figure 9b). The maximum von Mises equivalent stress of the steel tubes was 477 MPa (Figure 9c), which reached the ultimate strength of the steel.

(2) Hysteresis curves and the skeleton curve

**Figure 10:**
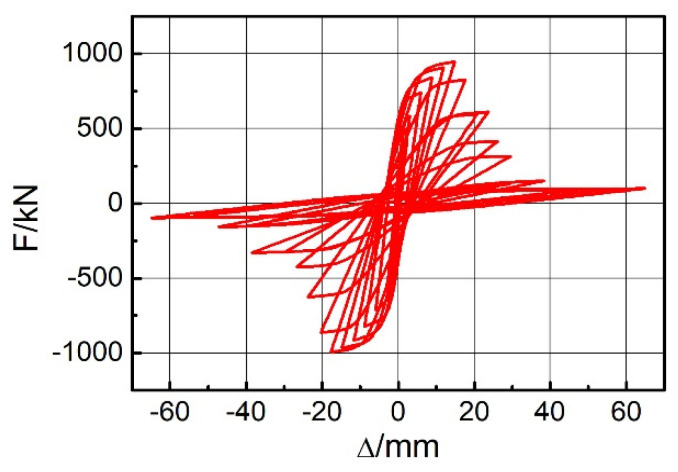
Hysteresis curves of the EDCW bearing capacity.

The hysteresis curves of the EDCW obtained from numerical simulation were butterfly shaped (Figure 10) and could be roughly divided into two groups. One group of hysteresis curves had a steep slope, corresponding to the concrete wall working together with the boundary steel tubes, and the other group had a gentle slope and was obtained after the failure of the concrete wall.

**Figure 11:**
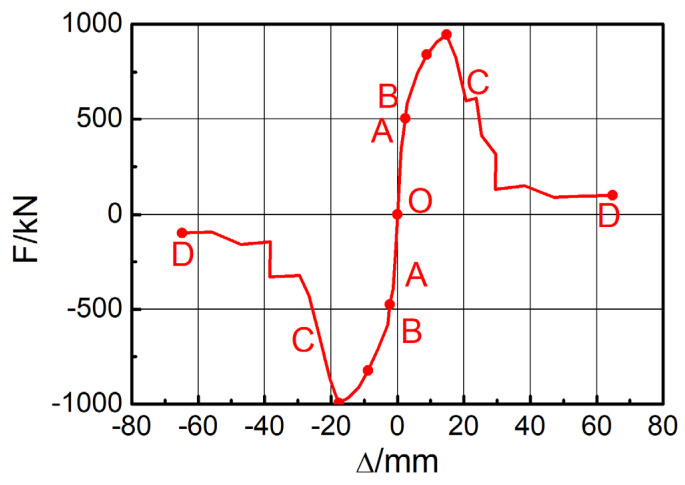
Skeleton curve. A—cracking point; B—yield point; C—peak point; D—point of destruction; O—zero.

The skeleton curve could visually reflect the variation patterns of the horizontal lateral stiffness and the bearing capacity of the structure under cyclic loading and was an important characterization of the seismic performance of the structure. Figure 10 shows the skeleton curve obtained from the numerical simulation, which can be divided into four development stages (the red curve in Figure 11).

Stage I: Section OA of the skeleton curve. In this stage, the drift ratio of the EDCW was less than 1/500, which corresponds to the initial stage of loading, when the EDCW was in the elastic deformation stage and had high stiffness.

Stage II: Section AB of the skeleton curve. In this stage, the drift ratio of the EDCW was between 1/500 and 1/200, the concrete wall gradually yielded, and its stiffness also decreased gradually but slightly. The load corresponding to point B was the bearing capacity of the EDCW at yield.

Stage III: Section BC of the skeleton curve. The drift ratio of the EDCW was between 1/200 and 1/130. In this stage, the damaged area of the concrete wall increased rapidly. After reaching point C, the concrete wall completely failed and lost its bearing capacity. Therefore, the corresponding load at point C was the peak bearing capacity of the EDCW.

Stage IV: Section CD of the skeleton curve. The drift ratio of the EDCW was between 1/130 and 1/27. In this stage, the load was entirely taken by the CFSTCs, and the stiffness decreased sharply. Upon reaching point D, the boundary CFSTCs failed, and the corresponding load was the bearing capacity of the EDCW at failure.

### 4.2. Seismic Performance of the Steel Frame Structure with EDCWs

#### 4.2.1. Simulation Scheme

The present study carried out a numerical simulation of cyclic loading tests on 16 steel frame structures to investigate their seismic performance. The 16 steel frames were divided into four groups. The first group consisted of five frames that had the same stiffness and the same installation location of the EDCW but different spans. The second group also included five frames that had the same span and the same stiffness but different installation locations of the EDCW. The third group consisted of another five frames that had the same span and the same installation location of the EDCW but different stiffnesses. The fourth group included only one frame without the EDCW. Table 4 lists the parameters of the 16 steel frame structures.

#### 4.2.2. Seismic Performance of the Frames

(1) Structural failure mode

**Figure 12:**
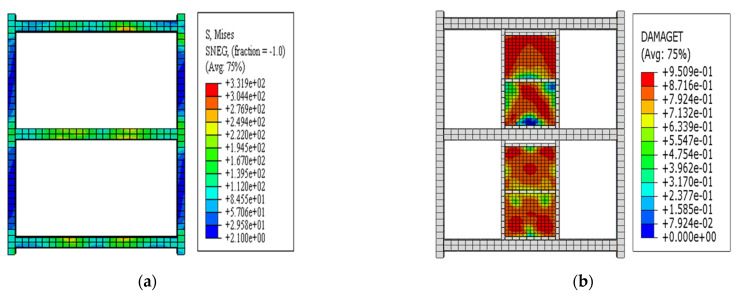
Failure modes of frame F2. (**a**) DAMAGET contour plot of the wall panel element; (**b**) von Mises stress contour plot of the frame.

This section uses frame F2 as an example to discuss the seismic performance of the steel frame structure with EDCWs. The cyclic loading test on F2 was simulated according to the loading protocol shown in Figure 5. As shown in Figure 12a, when loaded to a drift ratio of 1/30, the EDCWs failed. The yielded region of wall II2 was symmetrically distributed about the vertical centreline of the wall, with a large area accounting for 90.27% of the total area. The yielded region of wall II1 had an inverted V-shape, with an area of approximately 69.16% of the total area. The yielded region of wall I2 extended from the middle to the four corners, with an area accounting for 87.67% of the total area. The yielded region of wall I1 was W-shaped, with an area accounting for 84.26% of the total area. The total yielded area and total failure area of the EDCWs within the frame accounted for approximately 83.24% and 71.97% of the overall total area, respectively. At this moment, the maximum von Mises equivalent stress of the steel frame was 304.4 MPa (Figure 12b), which was less than the yield strength of steel, indicating that the steel frame still performed normally.

(2) Hysteresis curves and skeleton curves

**Figure 13:**
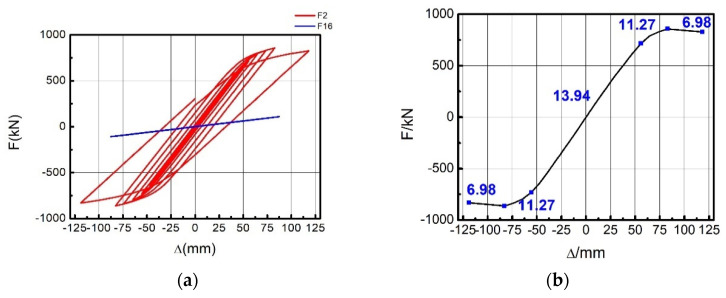
Hysteresis curves and the skeleton curve of frame F2. (**a**) Hysteresis curves of F2 and F16; (**b**) skeleton curve of F2.

The red line in Figure 13a is the hysteresis curve of F2 obtained from numerical simulation. The curve was full and spindle-shaped, indicating that the whole structure had a high plastic deformation capacity as well as good seismic performance and energy dissipation capacity. Figure 13b shows the skeleton curve of F2, from which the load–displacement curve of the frame included an elastic stage, an elastoplastic stage, and a failure stage. When the horizontal load was less than 715.60 kN, the skeleton curve was linear, with a stiffness of 13.94 kN/mm; when the horizontal load was between 715.60 kN and 857.69 kN, the curve entered a nonlinear stage with the stiffness decreasing to 11.27 kN/mm; and when the horizontal load exceeded 857.69 kN, the curve started to descend, with a stiffness of 6.98 kN/mm. The peak bearing capacity of the frame was 857.69 kN.

The blue line in Figure 13a is the hysteresis curve of the frame without the EDCW (F16). A comparison of the hysteresis curves of F2 and F16 revealed that the dissipated energy and bearing capacity of F2 were 174.24- and 7.8-fold greater than those of F16, respectively, indicating that the seismic performance of the steel frame structure was substantially improved by adding the EDCWs.

#### 4.2.3. Effects of Structural Design Parameters on the Seismic Performance of Frames

(1) Effect of the span ratio of the steel frame to the EDCW on seismic performance

**Figure 14:**
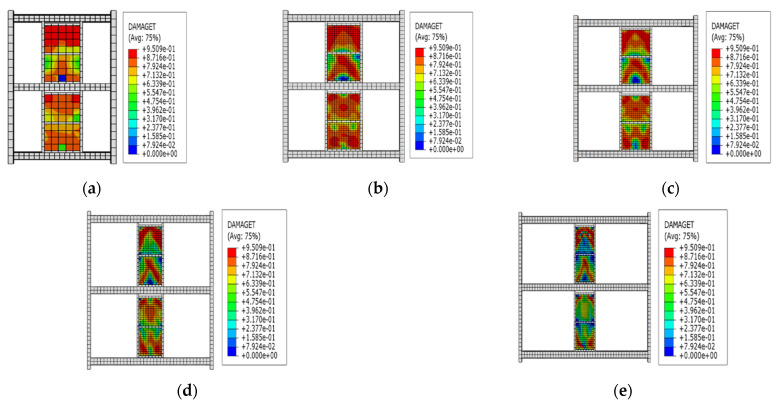
Stress contour plots of the first group of frames with the EDCWs at failure. (**a**) F1; (**b**) F2; (**c**) F3; (**d**) F4; and (**e**) F5.

The frames (F1 to F5) of the first group had a steel frame to EDCW span ratios of 2.4, 3, 3.6, 4.5, and 6, and the EDCWs were located in the middle of the frames. Figure 14a–e shows the stress contour plots of the EDCWs in the five frames when loaded to a drift ratio of 1/30. At this moment, all the EDCWs failed, but the failure area of the EDCWs decreased with an increasing span ratio and was 86.13%, 83.24%, 70.24%, 54.21%, and 43.31% of the total area for the five frames. Therefore, the extent of damage to the EDCWs decreased with an increasing span ratio.

**Figure 15:**
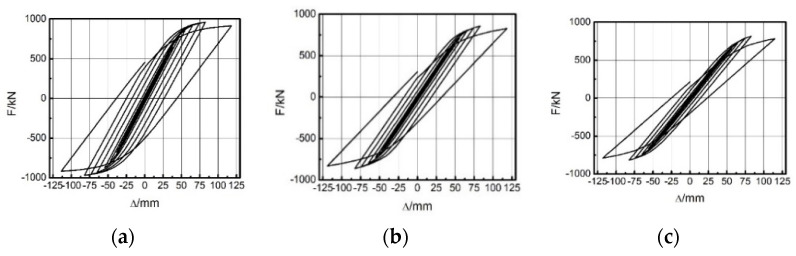

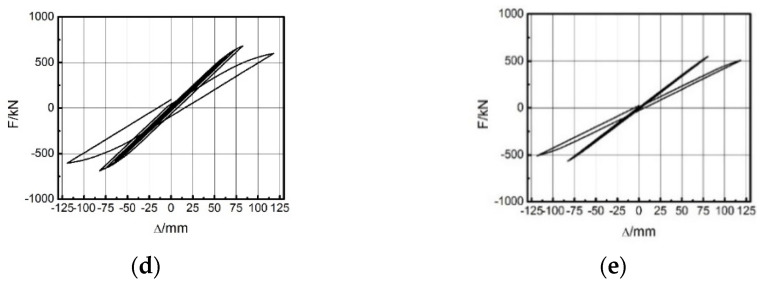
Hysteresis curves of the first group of frames. (**a**) F1; (**b**) F2; (**c**) F3; (**d**) F4; and (**e**) F5.

Different span ratios led to different energies dissipated by the EDCW. Figure 15a–e show the hysteresis curves of the five frames in the first group. As the span ratio increased, the hysteresis loop area gradually decreased, and thus, the dissipated energy gradually decreased (Table 5). In addition, the maximum bearing capacities of the five frames were 960.61 kN, 857.69 kN, 814.99 kN, 682.98 kN, and 548.83 kN, indicating that the bearing capacity of the frame decreased substantially with an increasing span ratio. Therefore, the seismic effectiveness of the EDCW increased with a decreasing span ratio.

(2) Effect of the installation location of the EDCW on the seismic performance of the steel frame

**Figure 16:**
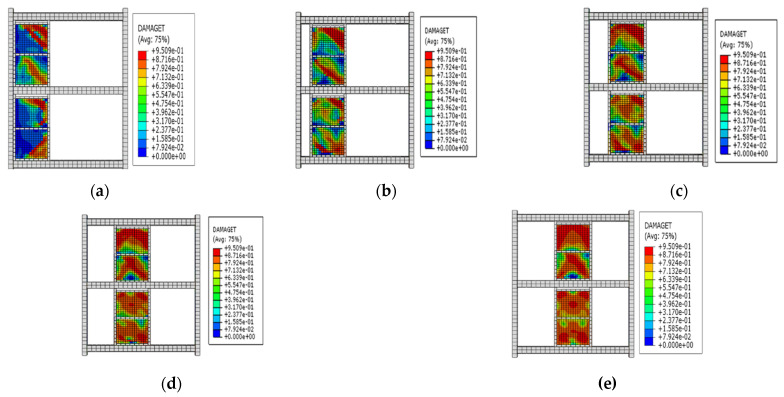
Stress contour plots of the EDCWs installed at different locations. (**a**) F6; (**b**) F7; (**c**) F8; (**d**) F9; and (**e**) F10.

The second group of frames (F6 to F10) had a net span of 4800 mm and the same span ratio of the frame to the EDCW, and the distance L between the vertical centreline of the EDCW and the left column of the frame was 820 mm, 1215 mm, 1610 mm, 2005 mm, and 2400 mm, respectively. Figure 16a–e shows the stress contour plots of the EDCWs in the five frames when loaded to a drift ratio of 1/30. At this drift ratio, the EDCWs had already failed, but the failure area of the EDCWs increased with increasing L and was 36.15%, 47.31%, 57.91%, 77.29%, and 83.24% of the total area, respectively. Therefore, the extent of damage to the EDCWs increased with increasing L, while the extent of the failure of the EDCWs was highest when the EDCWs were in the middle of the frame.

**Figure 17:**
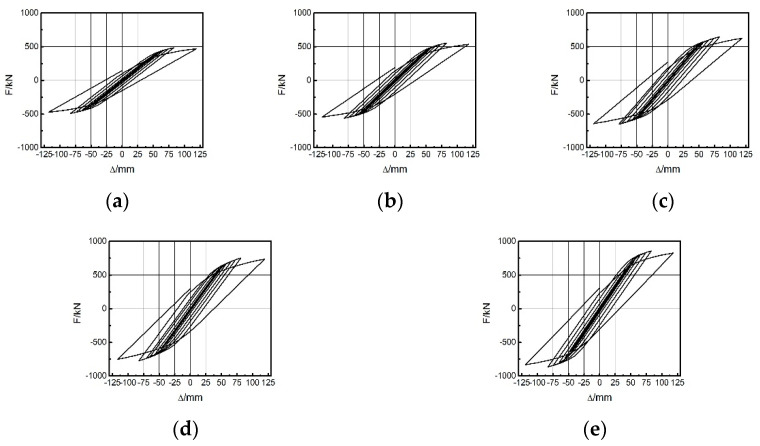
Hysteresis curves of the EDCWs installed at different locations. (**a**) F6; (**b**) F7; (**c**) F8; (**d**) F9; and (**e**) F10.

Figure 17a–e shows the hysteresis curves of the five frames; as L increased, the hysteresis loop area and thus the dissipated energy gradually increased (Table 6). The maximum bearing capacities of the five frames (F6 to F10) were 484.87 kN, 554.84 kN, 645.91 kN, 753.85 kN, and 857.69 kN, indicating that as the installation location of the EDCW moved from left to centre, the bearing capacity of the frame increased substantially. Therefore, the frame had the optimal seismic performance when the EDCWs were installed in the middle of the frame.

(3) Effect of steel frame stiffness on seismic performance

**Figure 18:**
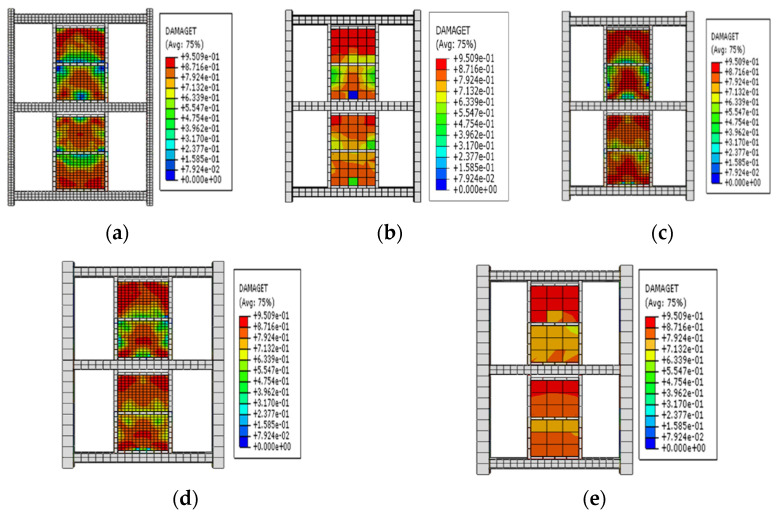
Stress contour plots of the EDCWs. (**a**) F11; (**b**) F12; (**c**) F13; (**d**) F14; and (**e**) F15.

The frames (F11 to F15) in the third group had different cross-sectional dimensions of steel beam columns but had the same construction dimensions and material properties of the EDCWs as well as the same distance of 2400 mm between the centreline of the EDCW and the left column of the frame. The cross-sectional dimensions and stiffness of the beams and columns gradually increased from F11 to F15. Figure 18a–e shows the stress contour plots of the EDCWs in the five frames when loaded to a drift ratio of 1/30. At this drift ratio, all EDCWs failed, but the failure area of the EDCWs increased with increasing stiffness of the steel frame and was 73.26%, 86.13%, 88.98%, 92.11%, and 97.61% of the total area, respectively. Therefore, the extent of damage to the EDCWs increased with the stiffness of the steel frame.

**Figure 19:**
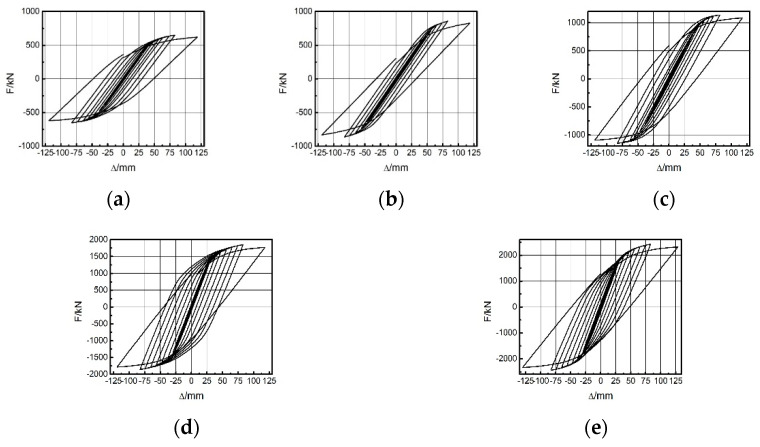
Hysteresis curves of the third group of frames: (**a**) F11; (**b**) F12; (**c**) F13; (**d**) F14; and (**e**) F15.

Figure 19a–e shows the hysteresis curves of the five frames. The figure shows that as the frame stiffness increased, the hysteresis loop area and hence the dissipated energy increased gradually (Table 7). The maximum bearing capacities of the five frames (F11 to F15) were 647.78 kN, 960.61 kN, 1137.23 kN, 1851.16 kN, and 2242.65 kN, indicating that as the stiffness of the steel frame increased, the bearing capacity of the frame increased substantially, and the energy dissipation capacity of the EDCW also increased.

### 4.3. Discussion

(1) An energy-dissipating composite wall (EDCW) was designed to improve the seismic performance of steel frame buildings.
(2) The EDCW was installed in a two-storey steel frame, and the seismic performance of the steel frame was analysed using the FEM. The results showed that the EDCW dissipated most of the seismic energy and thus substantially improved the seismic performance of the frame.

## 5. Conclusions

(1) A preliminary finite element model was developed to test the seismic performance of an energy-dissipating composite wall (EDCW), and the hysteretic curves obtained by numerical simulation were compared with the experimental hysteretic curves. The curves fit each other, and the development pattern was consistent, verifying the reliability of the finite element analysis;
(2) An EDCW that was assembled from concrete-filled steel tubular columns (CFSTCs) and concrete sheet walls were designed to improve the seismic performance of steel frame houses in rural areas. The seismic performance of the EDCW was obtained by reproducing the cyclic loading test on the EDCW using numerical simulations. The EDCW had a butterfly-shaped hysteresis curve. Before the concrete wall failed, the wall and frame worked together, resulting in a steep hysteresis curve; after failure of the concrete wall, the CFSTCs resisted the seismic load, leading to a gentle hysteresis curve;
(3) The seismic performance of a two-storey steel frame installed with EDCWs was analysed by using the finite element method to obtain the deformation and failure characteristics, hysteresis curves, and skeleton curves. The numerical simulation results showed that the EDCWs dissipated most of the seismic energy during earthquakes, and hence, the seismic performance of the steel frame was substantially improved by the installation of the EDCWs;
(4) The seismic performances of 16 frames were compared to investigate the effects of the span ratio of the steel frame to the EDCW, the installation location of the EDCW, and the stiffness of the steel frame on the seismic performance of the frame. With the increase in the span ratio, the extent of damage to the EDCW decreased, as did its seismic effectiveness. The farther the installation location of the EDCW was from the side column of the frame, the more energy was dissipated by the EDCW, and the best seismic performance was achieved when the EDCW was in the middle of the frame. As the stiffness of the steel frame increased, the extent of damage to the EDCW increased, as did its energy dissipation capacity;
(5) According to the seismic performance analysis of 16 trusses of frames, the component proposed in this paper could improve the overall stiffness of the frame and absorb most of the seismic energy under the seismic load, thus reducing the damage of the external frame to within a negligible range. Since the overall structure could still perform its original function, the cost of reconstruction after an earthquake could be reduced by replacing only the most severely stressed concrete wall sheet or replacing the whole internal component to keep the structure in use.

## Figures and Tables

**Table 1:** Mechanical parameters of the boundary components of the EDCW.

Steel Type	Steel Specifications	Yield Strength *f_y_* (MPa)	Ultimate Strength *f_u_* (MPa)	Elongation Δ (%)	Elastic Modulus *E* (MPa)	Thickness of the Steel Plate (mm)
Distribution rebar	Φ5	680	786	5.50	2.09 × 10^5^	-
Steel plate strip	40	309	467	25.27	2.11 × 10^5^	4
Steel boundary component	100 × 100	375	477	23.23	2.18 × 10^5^	4

**Table 2:** Mechanical parameters of the concrete wall.

Concrete Grade	Components Cement, Water, Fine Aggregate, Coarse Aggregate, Fly Ash, Mineral Powder	Compressive Strength *f_cu_* (MPa)	Elastic Modulus *E_c_* (MPa)
C40	1:0.49:2.28:2.28:0.21:0.21	41.15	3.15 × 10^4^

**Table 3:** Sections and material mechanical properties of the structural components of the steel frame.

Steel Type	Section Dimensions (mm)	Yield Strength *f*_y_ (MPa)	Ultimate Strength *f*_u_ (MPa)	Elongation δ (%)	Elastic Modulus *E_s_* (MPa)
I-beam	350 × 200 × 6 × 8	375	477	23.23	2.18 × 10^5^
Square steel tube	200 × 200 × 8	375	477	23.23	2.18 × 10^5^

**Table 4:** Parameters of the steel frame structures.

No.	Frame Span (m)	Frame Beam Section Dimensions (mm)	Frame Column Section Dimensions (mm)	EDCW Span (mm)	Distance from the Midpoint of the EDCW to Left Column (mm)	Span Ratio (Frame Span/EDCW Span)
F1	3840	350 × 200 × 6 × 8	200 × 200 × 8	1600	1920	2.4
F2	4800	350 × 200 × 6 × 8	200 × 200 × 8	1600	2400	3.0
F3	5760	350 × 200 × 6 × 8	200 × 200 × 8	1600	2880	3.6
F4	6720	350 × 200 × 6 × 8	200 × 200 × 8	1600	3360	4.2
F5	7680	350 × 200 × 6 × 8	200 × 200 × 8	1600	3840	4.8
F6	4800	350 × 200 × 6 × 8	200 × 200 × 8	1600	800	3.0
F7	4800	350 × 200 × 6 × 8	200 × 200 × 8	1600	1000	3.0
F8	4800	350 × 200 × 6 × 8	200 × 200 × 8	1600	1920	3.0
F9	4800	350 × 200 × 6 × 8	200 × 200 × 8	1600	3040	3.0
F10	4800	350 × 200 × 6 × 8	200 × 200 × 8	1600	2840	3.0
F11	3840	350 × 160 × 5 × 7	160 × 160 × 8	1600	1920	3.0
F12	3840	350 × 200 × 6 × 8	240 × 240 × 8	1600	1920	2.4
F13	3840	350 × 200 × 6 × 8	200 × 200 × 8	1600	1920	2.4
F14	3840	350 × 240 × 7 × 10	300 × 300 × 8	1600	1920	2.4
F15	3840	350 × 300 × 9 × 12	360 × 360 × 8	1600	1920	2.4
F16	4800	350 × 360 × 10 × 14	200 × 200 × 8	-	-	-

**Table 5:** Cumulative energy dissipated by the EDCWs.

Frame ID	Span Ratio	Dissipated Energy (kN·mm)	Ratio to That of F5
F1	0.8 L	13.2451 × 10^4^	16.1415
F2	L	9.0388 × 10^4^	11.0154
F3	1.2 L	6.2363 × 10^4^	7.60001
F4	1.5 L	2.9012 × 10^4^	3.5356
F5	2 L	0.8205 × 10^4^	1.0000

**Table 6:** Cumulative dissipated energies of the EDCWs.

Frame ID	Distance *L* from the Centreline of the EDCW to the Left Column of the Frame (mm)	Dissipated Energy (kN·mm)	Ratio to That of F6
F6	820	4.4595 × 10^4^	1.0000
F7	1215	5.5622 × 10^4^	1.2473
F8	1610	7.4544 × 10^4^	1.6716
F9	2005	8.6705 × 10^4^	1.9443
F10	2400	9.0388 × 10^4^	2.0269

**Table 7:** Cumulative dissipated energies of the EDCWs.

Frame ID	Cross-Sectional Expansion Multiplier	Dissipated Energy (kN·mm)	Ratio to That of F11
F11	0.8	9.8993 × 10^4^	1.0000
F12	1	13.2451 × 10^4^	1.3380
F13	1.2	16.3053 × 10^4^	1.6471
F14	1.5	26.6152 × 10^4^	2.6886
F15	1.8	38.0334 × 10^4^	3.8420

## Data Availability

Not applicable.
